# Post-Tuberculosis Sequelae and Active Tuberculosis in Lung Cancer: Imaging Patterns and Clinical Associations—A Retrospective Single-Center Cohort Study

**DOI:** 10.3390/jcm15135063

**Published:** 2026-06-29

**Authors:** Cristina Cioti, Cristina Tocia, Nejla Dervis, Ioan Anton Arghir, Simona Buligan, Gabriela Fricatel, Mihaela Pundiche, Oana Cristina Arghir

**Affiliations:** 1 Internal Medicine Department, “Sf. Apostol Andrei” Emergency County Hospital, 145 Tomis Blvd., 900591 Constanta, Romania; cristina.cioti@365.univ-ovidius.ro; 2 School of Medicine, “Ovidius” University of Constanta, 1 University Street, 900470 Constanta, Romania; nejla.dervis@365.univ-ovidius.ro (N.D.); arghir_oana@yahoo.com (O.C.A.); 3 Pneumology, Municipal Hostpital, 820195 Tulcea, Romania; ionut_arghir93@yahoo.com; 4 School of Medicine, Carol Davila University of Medicine and Pharmacy, 8 Eroii Sanitari Boulevard, 050474 Bucharest, Romania; culacsiz.simona91@yahoo.com; 5 Oncology Department, “Sf. Apostol Andrei” Emergency County Hospital, 145 Tomis Blvd., 900591 Constanta, Romania; 6 Surgical Disciplines, “Ovidius” University of Constanta, 1 University Street, 900470 Constanta, Romania; mihaelapundiche@yahoo.com; 7 Clinical Pneumology, Hospital of Constanta, 40 Sentinelei Str., 900002 Constanta, Romania

**Keywords:** pulmonary tuberculosis, lung cancer, pulmonary imaging, post-tuberculosis sequelae, thoracic computed tomography, respiratory symptoms, lung malignancy

## Abstract

**Background:** Tuberculosis-related pulmonary changes may overlap radiologically and clinically with lung cancer, complicating diagnostic interpretation, staging, and therapeutic planning. This study evaluated the relationship between tuberculosis status, thoracic imaging patterns, and clinical characteristics in patients diagnosed with lung cancer. **Methods:** A retrospective cohort study was conducted between February 2020 and December 2025 at the Clinical Pneumophtisiology Hospital, Constanța, Romania. A total of 620 patients with lung cancer were analyzed. Patients were classified into three groups: no tuberculosis, post-tuberculosis sequelae, and active tuberculosis. Demographic, clinical, laboratory, histopathological, functional, and radiological variables were assessed. Associations between tuberculosis status and imaging findings were evaluated using chi-square testing, effect-size analysis, and multinomial logistic regression. **Results:** Post-tuberculosis sequelae were identified in 337 patients (54.4%), active tuberculosis in 51 patients (8.2%), and no tuberculosis-related disease in 232 patients (37.4%). Adenocarcinoma was the most frequent histological type, occurring in 359 patients (57.9%). Significant associations with tuberculosis status were observed for fibrotic/interstitial or bronchial changes, infectious-inflammatory changes, cavitary/destructive lesions, atelectatic/retractile changes, pulmonary opacities, pleural involvement, and mediastinal/hilar adenopathy. The strongest effects were found for fibrotic/interstitial changes, infectious-inflammatory changes, and cavitary/destructive lesions. In regression analysis, active tuberculosis was most strongly associated with infectious-inflammatory changes, cavitary lesions, pulmonary opacities, and fibrotic abnormalities, while post-tuberculosis sequelae were mainly associated with chronic fibrotic and structural pulmonary changes. **Conclusions:** Tuberculosis-related abnormalities frequently coexist with lung cancer and may mimic or obscure malignant findings. Recognition of these overlapping patterns is essential for accurate radiological interpretation and individualized clinical management.

## 1. Introduction

Lung cancer and pulmonary tuberculosis remain major global public health problems and frequently coexist in regions with high respiratory disease burden [1]. Both conditions may present with overlapping clinical manifestations and similar thoracic imaging abnormalities, creating substantial diagnostic challenges in daily clinical practice [2]. Chronic cough, dyspnea, hemoptysis, constitutional symptoms, pulmonary nodules, cavitary lesions, fibrosis, and consolidative changes may be encountered in both pulmonary malignancy and tuberculosis, often delaying accurate diagnosis and appropriate therapeutic intervention [3]. Recent evidence has emphasized that cavitary and destructive pulmonary lesions may closely mimic malignant disease, particularly in patients with previous tuberculosis-related structural lung damage [4,5,6].

Advanced imaging techniques have been increasingly explored to improve the differentiation between pulmonary tuberculosis and lung cancer [7]. Luo et al. demonstrated the potential value of multi-time-point ^18F-FDG PET/CT imaging in distinguishing lung cancer from tuberculosis-related lesions, although significant overlap in metabolic activity continues to limit diagnostic specificity [8]. Similarly, Wei et al. reported that radiomics combined with conventional CT imaging features may improve the distinction between mass-like tuberculosis and pulmonary malignancy, highlighting the importance of integrated radiological assessment [9]. In parallel, recent European recommendations regarding lung cancer screening have underlined the growing role of imaging-based early detection strategies in high-risk populations with chronic pulmonary disease [10].

Patients with chronic respiratory comorbidities represent a particularly vulnerable population in whom overlapping inflammatory, infectious, and neoplastic pulmonary changes may coexist [11]. Lung cancer screening studies have shown that chronic pulmonary abnormalities substantially influence radiological interpretation and diagnostic pathways [12,13,14,15]. Moreover, tuberculosis itself may simulate malignant pulmonary lesions, further complicating clinical evaluation and radiological staging [16].

To our knowledge, few studies have simultaneously evaluated radiological overlap patterns, respiratory symptom burden, and functional impairment across active TB, post-TB sequelae, and lung cancer in a large hospitalized European cohort. The primary aim of this study was to evaluate radiological overlap patterns between pulmonary tuberculosis and lung cancer and identify imaging predictors independently associated with active or previous TB in patients with lung malignancy.

## 2. Materials and Methods

### 2.1. Study Framework and Patient Selection

This retrospective cohort study was performed at the Clinical Pneumophtisiology Hospital, Constanța, Romania. Data collection was conducted from February 2020 to December 2025. All patients included had complete follow-up data available at the time of database closure. The December 2025 end date reflects the final patient admission included in the dataset.

We screened for the eligibility of all consecutive patients with documented LC admitted during the study interval. Histopathological confirmation was the primary diagnostic standard. In cases where biopsy was technically unfeasible due to poor performance status, advanced disease, or inconclusive bronchoscopy, the diagnosis was supported by multidisciplinary radiological and clinical evaluation, consistent with institutional practice. We included patients who had sufficient demographic, clinical, imaging, and oncological data.

We classified TB-related status as follows: no TB, post-TB sequelae, and active TB.

We excluded patients who presented uncertain TB classification, duplicate medical records, incomplete essential documentation, or exclusively benign pulmonary pathology. We also excluded cases with partially incomplete radiological or oncological information.

Due to the retrospective nature of the study and variability in historical record availability over the five-year study period, systematic microbiological confirmation of prior tuberculosis could not be obtained for all patients in the post-TB sequelae group. Classification was based on the presence of characteristic structural imaging abnormalities consistent with established radiological criteria for post-TB lung damage, interpreted in conjunction with available clinical history and prior medical documentation.

**Figure 1:**
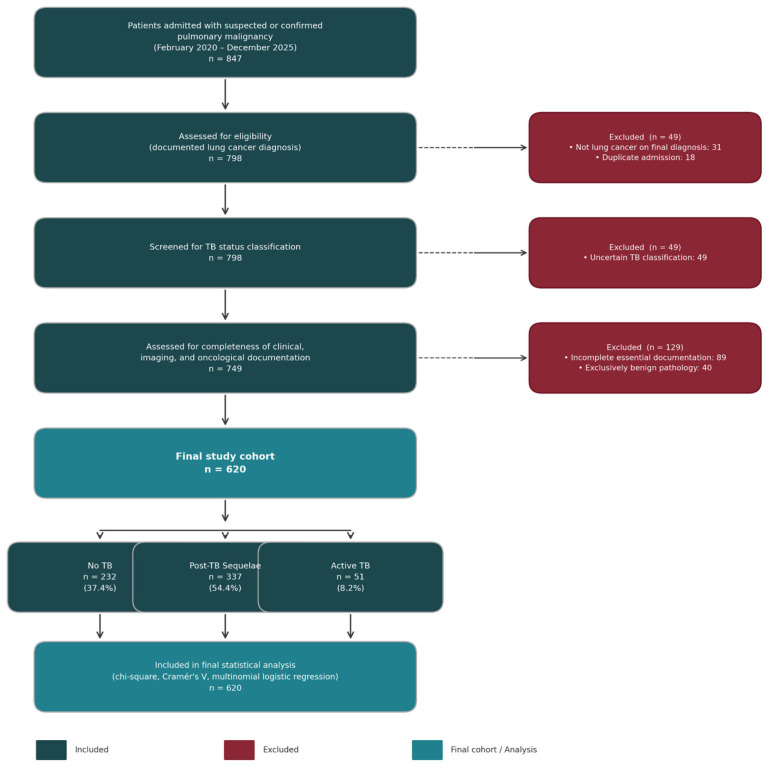
STROBE diagram for the study cohort.

A total of 227 patients were excluded from the initial screening and eligibility process: 49 due to uncertain TB classification, 18 due to duplicate admissions, 89 due to incomplete essential clinical, imaging, or oncological documentation, 40 due to exclusively benign pulmonary pathology, and 31 because the final diagnosis was not lung cancer. The final cohort included 620 patients (Figure 1).

### 2.2. Clinical and Functional Assessment

Clinical information was extracted retrospectively from hospital archives and electronic medical records. We decided that we will evaluate the demographic data, smoking status, pack-year exposure, oxygen therapy requirements, respiratory failure, COPD diagnosis, GOLD stage, cardiovascular and metabolic comorbidities, respiratory symptomatology, and physical examination findings. For respiratory manifestations, we analyzed cough with or without expectoration, dyspnea, hemoptysis, thoracic pain, digestive symptoms, and constitutional or inflammatory manifestations. We also added the spirometry patterns (categorized as normal, obstructive, restrictive, mixed, or nonspecific ventilatory dysfunction). Histopathological data included adenocarcinoma, squamous cell carcinoma, small cell carcinoma, NSCLC-unspecified, and other malignant or suspicious pathological categories. We classified tumor stage as early-stage or advanced-stage disease.

### 2.3. Radiological Evaluation and Tuberculosis Classification

The main imaging modality was computed tomography, but where this was not available, we used chest radiography and archived imaging reports.

The radiological analysis focused on the presence of:

- nodular lesions or metastatic dissemination,
- mediastinal or hilar adenopathy,
- pleural involvement,
- emphysematous or chronic obstructive changes,
- pulmonary opacities or condensation,
- pulmonary tumor masses,
- atelectatic or retractile changes,
- fibrotic/interstitial or bronchial abnormalities,
- cavitary or destructive lesions,
- infectious-inflammatory changes.

We defined post-TB sequelae by looking at structural pulmonary abnormalities associated with previous tuberculosis-related damage (apical fibrotic scars, fibronodular lesions, calcified granulomas, traction bronchiectasis, pleural thickening, residual cavitary lesions, parenchymal distortion, volume loss, and calcified mediastinal lymph nodes). These criteria are consistent with published radiological definitions of post-TB lung damage. Each case was reviewed and classified by a multidisciplinary team combining radiological assessment with clinical history and available prior medical documentation.

### 2.4. Statistical and Exploratory Analysis

For the statistical analysis, we decided to use IBM SPSS Statistics version 30.0 (IBM Corp., Armonk, NY, USA). We expressed the continuous variables as mean ± standard deviation with 95% confidence intervals, and we reported the categorical variables as absolute frequencies and percentages.

We evaluated associations between TB status and radiological or clinical variables using Pearson’s Chi-square test. Additionally, we identified the effect size using Cramer’s V coefficient. Thus, we were able to quantify the magnitude of association between TB-related categories and thoracic imaging abnormalities.

Next, we performed a multinomial logistic regression analysis in order to identify independent imaging predictors associated with tuberculosis status. For this, we used the no-TB category as the reference group, and we calculated the odds ratios (ORs) with 95% confidence intervals (CIs). Statistical significance was established at *p* < 0.05.

Spirometry patterns were evaluated descriptively; spirometric data were not included as predictors in the regression model. Therefore, patients with missing spirometry values (*n* = 22, included in the ‘missing/not performed’ category) were not subject to listwise exclusion from regression analyses, and all 620 patients were included in the regression model.

We acknowledge that the active TB subgroup (*n* = 51) yields an events-per-variable ratio of approximately 3.9, below the recommended threshold of 10 for stable regression estimates. The model is therefore designated as exploratory, and the results should not be used for external prediction without validation in an independent cohort.

### 2.5. Ethical Approval

The study was conducted according to the ethical principles of the Declaration of Helsinki. Ethical approval was obtained from the Ethics Committee of the Clinical Pneumophtisiology Hospital Constanța, Romania (Approval No. 473/11 February 2020).

## 3. Results

### 3.1. Population Characteristics

The study population consisted of 620 patients evaluated for demographic, clinical, and laboratory characteristics (Table 1). We performed descriptive statistical analysis for continuous variables using mean, standard deviation (SD), and 95% confidence intervals (CI) for the mean, while categorical variables were summarized using absolute frequencies and percentages. The analyzed parameters included demographic distribution, smoking status, respiratory function, oxygen therapy requirements, and routine laboratory investigations (Table 1).

The mean age was 67 ± 10 years. Male patients represented the majority of the population (56.9%), while 43.1% were female. Most participants originated from urban areas (59.8%), compared with 40.2% from rural settings. Smoking history was highly prevalent, being documented in 69.4% of patients, with a mean pack-year index of 40 ± 25.

Laboratory investigations demonstrated evidence of chronic inflammation and systemic impairment within the cohort. Mean Hb levels were 9 ± 3 g/dL, while the average WBC count was 10 ± 4 × 10^3^/µL and the platelet count reached 323 ± 133 × 10^3^/µL. Inflammatory activity was reflected by elevated ESR values, with a mean of 50 ± 36 mm/h. Metabolic parameters revealed a mean fasting blood glucose level of 116 ± 44 mg/dL. Liver function tests showed mean AST and ALT levels of 30 ± 17 U/L and 24 ± 26 U/L, respectively. Renal function parameters included a mean urea value of 47 ± 27 mg/dL and serum creatinine of 1.09 ± 0.57 mg/dL.

Respiratory and cardiovascular assessment highlighted significant functional impairment among patients. Mean peripheral oxygen saturation (SpO_2_) was 91 ± 9%, indicating frequent hypoxemia. The average systolic blood pressure was 133 ± 20 mmHg, while the mean heart rate reached 80 ± 29 beats/minute.

### 3.2. Histopathological, Tuberculosis-Related, and Functional Characteristics

Table 2 summarizes the distribution of TB status, histopathological diagnoses, cancer stage, pulmonary functional impairment, and major respiratory and cardiovascular comorbidities within the study cohort. Post-TB sequelae were identified in 337 patients, representing 54.4% of the cohort. Active TB was present in 51 patients, corresponding to 8.2%, while 232 patients had no evidence of current or previous TB-related pulmonary disease.

Adenocarcinoma was the most frequent histopathological diagnosis, identified in 359 patients (57.9%). NSCLC, unspecified, was recorded in 89 patients (14.4%), followed by squamous cell carcinoma in 71 patients (11.5%) and small cell carcinoma in 32 patients (5.2%). Advanced-stage disease was present in 375 patients (60.5%), while 245 patients (39.5%) had early-stage disease.

COPD was documented in 379 patients (61.1%). Among COPD-related classifications, GOLD III and GOLD IV were the most frequent advanced categories, affecting 122 patients (19.7%) and 111 patients (17.9%), respectively. Spirometry data were interpretable or classifiable in 598 records. Within this subgroup, obstructive dysfunction was the most common pattern, identified in 179 patients (29.9%), followed by mixed dysfunction in 139 patients (23.2%). Respiratory failure was present in 268 patients (43.2%), hypertension in 325 patients (52.4%), and heart failure in 178 patients (28.7%).

**Figure 2:**
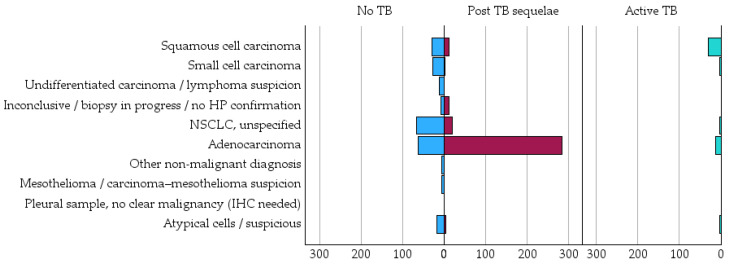
Distribution of histopathological subtypes according to TB status. No TB—patients without a tuberculosis-related disease; Post-TB—post-tuberculosis sequelae; Active TB—active tuberculosis; NSCLC—non-small cell lung cancer, unspecified; SCC—squamous cell carcinoma; SCC small cell—small cell carcinoma.

Figure 2 illustrates the distribution of histopathological lung cancer subtypes across patients without TB, with post-TB sequelae, and with active TB.

Adenocarcinoma represented the predominant histopathological subtype in all tuberculosis categories, with the highest frequency observed among patients with post-TB sequelae. NSCLC-unspecified and squamous cell carcinoma were also identified across all groups, while small cell carcinoma and other rare histopathological diagnoses were less frequent.

**Figure 3:**
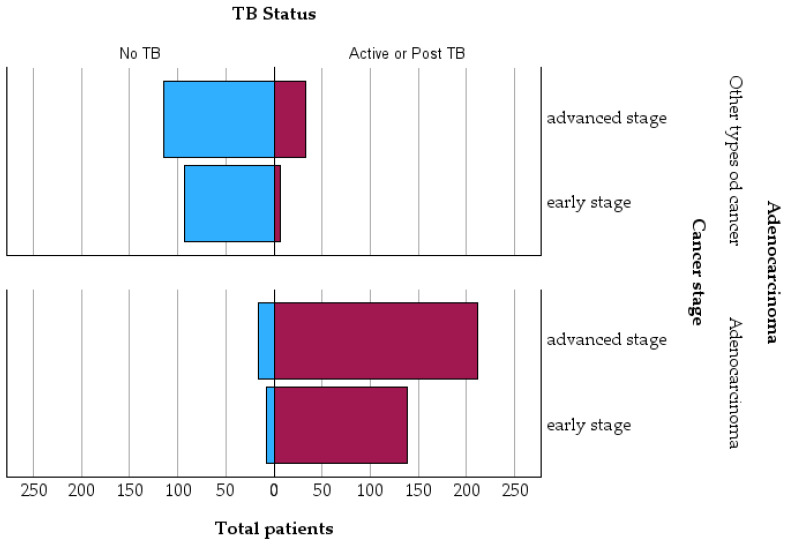
Association between tuberculosis status, adenocarcinoma, and cancer stage.

In Figure 3, we show the distribution of cancer stages according to tuberculosis status and adenocarcinoma.

Advanced-stage disease was more frequent than early-stage disease among patients with adenocarcinoma, particularly in individuals with active or post-TB status.

In contrast, other histopathological cancer subtypes showed lower frequencies and a less pronounced distribution across oncological stages.

### 3.3. Imaging Characteristics According to Tuberculosis Status and Histopathological Diagnosis

Radiological findings differed across TB-related categories. Among patients with post-TB sequelae, the most frequent abnormalities were fibrotic/interstitial or bronchial changes in 230 patients (68.2%), pulmonary tumor or expansive masses in 205 patients (60.8%), emphysematous/chronic obstructive changes in 190 patients (56.4%), nodular lesions or metastatic dissemination in 180 patients (53.4%), and pulmonary opacities or condensation in 165 patients (49.0%). Cavitary or destructive lesions were present in 105 patients (31.2%), while infectious-inflammatory changes were present in 92 patients (27.3%).

Patients with active TB showed a different radiological profile. Infectious-inflammatory changes were present in 39 patients (76.5%), cavitary or destructive lesions in 36 patients (70.6%), pulmonary opacities or condensation in 34 patients (66.7%), pulmonary tumor or expansive masses in 30 patients (58.8%), and fibrotic/interstitial or bronchial changes in 29 patients (56.9%). Mediastinal or hilar involvement with adenopathy was observed in 20 patients (39.2%).

Among patients with adenocarcinoma, pulmonary tumors or expansive masses were present in 241 patients (67.1%), nodular lesions or metastatic dissemination in 208 patients (58.0%), emphysematous/chronic obstructive changes in 191 patients (53.2%), pulmonary opacities or condensation in 164 patients (45.7%), and fibrotic/interstitial or bronchial changes in 154 patients (42.9%). Infectious-inflammatory changes were present in 120 adenocarcinoma cases (33.4%), while cavitary/destructive lesions were present in 101 cases (28.1%).

### 3.4. Clinical Manifestations According to Tuberculosis Status and Cancer Stage

Smoking exposure was frequent across all TB categories and was highest among patients with active TB, where it was documented in 45 of 51 patients (88.2%). Obstructive ventilatory dysfunction was also most frequent in the active TB group, affecting 31 patients (60.8%). Dyspnea was present in 42 active TB patients (82.4%), while cough with or without expectoration was present in 39 active TB patients (76.5%).

Across oncological stages, dyspnea increased with disease advancement. It was present in 12 patients with stage I disease (14.8%), 47 with stage II disease (28.7%), 139 with stage III disease (78.1%), and 169 with stage IV disease (85.8%). Oxygen therapy was required in 42 stage I patients (51.9%), 56 stage II patients (34.1%), 71 stage III patients (39.9%), and 79 stage IV patients (40.1%). Crackles were more frequent in active TB patients (45.1%) and in stage IV disease (37.6%). Cough remained common across all stages, affecting 67.9% of stage I, 64.0% of stage II, 60.1% of stage III, and 63.5% of stage IV patients.

### 3.5. Association Between Tuberculosis Status and Thoracic Imaging Findings

Pearson chi-square testing showed that the strongest associations with tuberculosis status were observed for fibrotic/interstitial or bronchial changes, infectious-inflammatory changes, cavitary/destructive lesions, and atelectatic/retractile changes (Table 3). Pulmonary opacities or condensation, pleural involvement, and mediastinal/hilar adenopathy also differed significantly across tuberculosis categories. In contrast, nodular lesions/metastatic dissemination, emphysematous/chronic obstructive changes, and pulmonary tumor or expansive masses did not show statistically significant differences across TB-status groups, indicating that these findings were broadly distributed across the cohort rather than specific to tuberculosis status.

**Figure 4:**
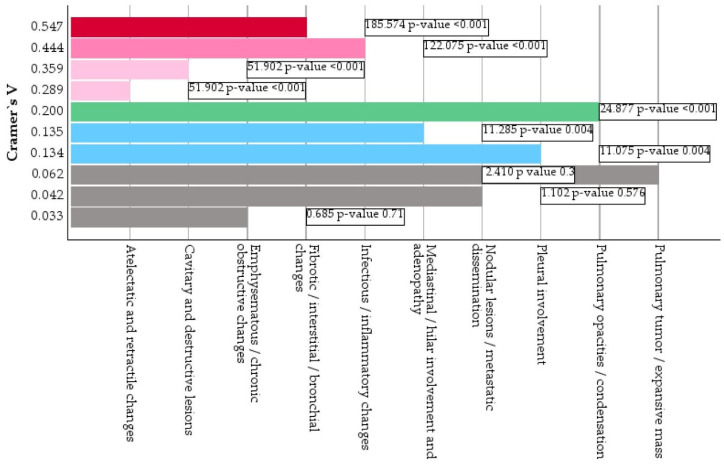
Comparative distribution of association strengths between radiological findings and study groups based on Cramer’s V analysis.

Figure 4 demonstrates the comparative strength of associations between radiological findings and the analyzed study groups. The strongest association was observed for fibrotic/interstitial/bronchial changes (V = 0.547, *p* < 0.001), followed by infectious/inflammatory changes (V = 0.444, *p* < 0.001) and cavitary/destructive lesions (V = 0.359, *p* < 0.001), indicating substantial differences between groups for these imaging patterns. Atelectatic/retractile changes also showed a moderate association (V = 0.289, *p* < 0.001). In contrast, nodular lesions, emphysematous changes, and pulmonary tumor masses demonstrated negligible associations and lacked statistical significance, suggesting limited discriminatory value between the evaluated groups.

### 3.6. Multinomial Logistic Regression Analysis

**Figure 5:**
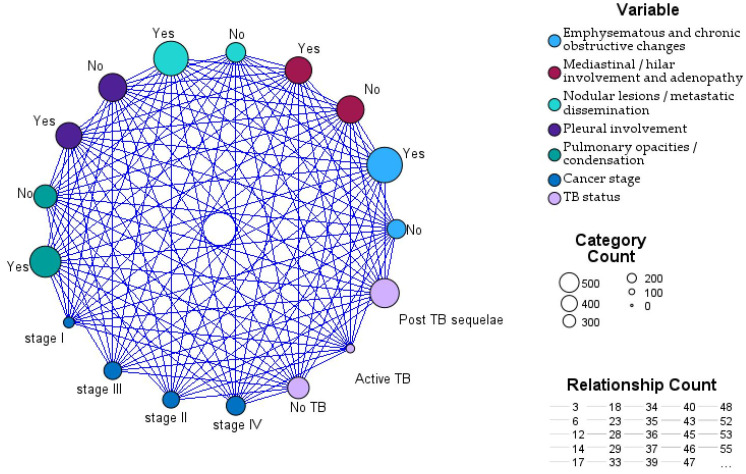
Network visualization of associations between tuberculosis status and radiological imaging findings.

We performed a multinomial logistic regression to identify independent predictors associated with post-TB sequelae and active TB, using the no-TB group as the reference category. The model included clinical, demographic, laboratory, and radiological variables that were structurally independent of the TB-status classification criteria. The predictors entered into the model were age, sex, smoking status, pack-year index, COPD diagnosis, respiratory failure, cancer stage, dyspnea severity, cough, hemoptysis, Hb, WBC, PLT, ESR, nodular lesions or metastatic dissemination, mediastinal or hilar involvement, pleural involvement, emphysematous or chronic obstructive changes, and pulmonary opacities or condensation. In Table 4, we present the full parameter estimates (Figure 5).

Emphysematous and chronic obstructive changes demonstrated extensive connectivity across the network, appearing among the most frequently present findings (large “Yes” node), with broad links to all three TB-status categories and all cancer stages. Pulmonary opacities or condensation and nodular lesions or metastatic dissemination showed similarly widespread interconnections, indicating that these radiological patterns were distributed across the cohort regardless of TB status. Mediastinal or hilar involvement and adenopathy displayed a more concentrated pattern of connections, with its “Yes” node linked particularly to active TB and post-TB sequelae categories. Pleural involvement showed a comparable distribution, with presence more strongly represented in post-TB sequelae.

Regarding cancer stage, stage IV and stage III nodes exhibited the highest relationship counts within the network, reflecting the predominance of advanced-stage disease in this cohort and its co-occurrence with multiple radiological abnormalities across all TB categories. Stage I and stage II nodes were smaller and less densely connected, consistent with their lower frequency in the cohort.

The results of this study demonstrated a substantial radiological and clinical overlap between pulmonary tuberculosis and lung cancer within the analyzed cohort. Structural pulmonary abnormalities were frequently identified across all three TB-status categories and across multiple oncological stages. Statistical analyses confirmed significant associations between several imaging findings and tuberculosis status, while the network visualization further supports the presence of interconnected radiological patterns rather than isolated manifestations.

## 4. Discussion

The present study demonstrated a substantial radiological and clinical overlap between pulmonary tuberculosis and lung cancer. Post-tuberculous structural sequelae represented the predominant TB-related category within the cohort, while active tuberculosis was identified in a smaller subgroup of patients. Multiple thoracic imaging abnormalities commonly associated with malignancy were frequently encountered in both active and previous tuberculosis-related disease. Similar observations have been reported in previous studies showing important morphological and metabolic overlap between pulmonary tuberculosis and lung malignancy, particularly in patients presenting with cavitary lesions, fibrosis, pulmonary nodules, or consolidative changes [17,18,19].

Our analyses demonstrated significant associations between TB status and the majority of evaluated radiological variables. Among the radiological findings assessed using Pearson chi-square testing, nodular lesions or metastatic dissemination (*p* = 0.576), emphysematous or chronic obstructive changes (*p* = 0.71), and pulmonary tumor or expansive masses (*p* = 0.30) did not show statistically significant differences across TB-status groups, indicating that these findings were broadly distributed across the cohort regardless of tuberculosis history.

The multinomial logistic regression model included only variables that were structurally independent of the TB-status classification criteria.

Smoking exposure was common across all tuberculosis and cancer-stage categories. Dyspnea, chronic cough, expectoration, thoracic pain, and constitutional symptoms were frequently encountered regardless of TB status. Overlapping respiratory symptom profiles have previously been described in patients with pulmonary TB and LC [20].

Previous imaging-based studies also reported that chronic structural pulmonary damage and inflammatory changes may obscure or delay recognition of malignant disease, particularly in regions with elevated tuberculosis burden [21,22,23].

In the current cohort, pleural involvement demonstrated a statistically significant association with TB status (χ^2^ = 11.075, *p* = 0.004, Cramér’s V = 0.134) and was independently associated with post-TB sequelae in the multivariable model (OR = 2.39, 95% CI 1.20–4.74, *p* = 0.013). It is important to emphasize that distinguishing a TB-related pleural effusion from a malignant effusion on imaging alone is diagnostically challenging, as both can present as unilateral exudates with overlapping radiological features, and histological or microbiological confirmation is often required for definitive characterization.

Patients with simultaneous active tuberculosis and lung cancer face compounded clinical challenges: diagnostic delays attributable to symptom overlap (hemoptysis, weight loss, respiratory symptoms), potential drug–drug interactions between anti-tuberculous agents and chemotherapy or targeted therapies, immunosuppression, and reduced performance status. Published evidence suggests shorter overall survival in patients with concurrent TB and lung cancer compared with lung cancer alone. In the current cohort, patients in the active TB group exhibited the highest rates of smoking history (88.2%), obstructive spirometric dysfunction (60.8%), and dyspnea (82.4%), consistent with a clinically more complex disease profile. In the multivariable model, male sex (OR = 18.52, 95% CI 6.67–50.00, *p* < 0.001) and smoking status (OR = 12.05, 95% CI 3.24–45.45, *p* < 0.001) were the strongest independent predictors of active TB classification. Survival data were not collected in this retrospective study. The heterogeneous radiological patterns identified in our cohort further support the need for integrated diagnostic approaches that combine imaging interpretation with clinical, functional, microbiological, and molecular evaluation [24,25].

The events-per-variable ratio of approximately 3.9 for the active TB outcome group (n = 51) falls below the widely recommended threshold of 10 required for stable logistic regression parameter estimates.

Several limitations of the present study should be acknowledged. The relatively high prevalence of post-tuberculous structural abnormalities reflects the characteristics of the study population. The patients originated from a tertiary referral pulmonology and pneumophysiology center.

The classification of post-tuberculous sequelae was based on characteristic structural imaging abnormalities interpreted together with compatible clinical history and available prior medical documentation. However, due to the retrospective design and incomplete historical microbiological records, systematic confirmation of previous tuberculosis could not be obtained in all patients included in the post-TB category.

The relatively small active TB subgroup (n = 51) relative to the number of predictors in the regression model raises concerns about overfitting; the model should be considered exploratory and validated in an independent cohort before being used for prediction.

Body mass index was not systematically available due to incomplete anthropometric documentation in the retrospective records and could not be included in the analyses.

Additionally, a subset of patients (n = 20 inconclusive/pending biopsy; n = 6 non-malignant; n = 25 atypical/suspicious cytology) lacked definitive histopathological confirmation of malignancy. These cases were included based on multidisciplinary clinical and radiological consensus at a tertiary referral center. This may introduce a degree of diagnostic uncertainty and should be considered when interpreting the results.

A systematic count of patients in the post-TB group with versus without prior microbiological confirmation of tuberculosis could not be obtained from retrospective records; future prospective studies should address this through standardized microbiological documentation protocols.

Finally, isolated emphysematous changes, smoking-related fibrosis, nonspecific chronic interstitial abnormalities, or COPD-associated structural alterations were not independently classified as post-tuberculous sequelae unless they were associated with imaging patterns considered characteristic of previous tuberculosis.

## 5. Conclusions

This study highlights the substantial radiological and clinical overlap between tuberculosis-related pulmonary abnormalities and lung cancer. Post-tuberculosis sequelae were frequent in this cohort, while active tuberculosis was less common but strongly associated with inflammatory, cavitary, and destructive imaging patterns. Chronic fibrotic, interstitial, and bronchial changes were the most characteristic findings in patients with previous tuberculosis, reflecting persistent structural lung damage that may complicate the interpretation of thoracic imaging in oncological settings.

We believe that tuberculosis status should be considered when evaluating patients with lung cancer, particularly in regions where tuberculosis remains prevalent or where post-tuberculosis lung disease is common. Cavitary lesions, pulmonary opacities, mediastinal or hilar adenopathy, and fibrotic abnormalities may mimic tumor progression, obscure malignant lesions, or contribute to diagnostic uncertainty. Therefore, careful correlation between imaging findings, clinical history, microbiological data, histopathology, and functional respiratory assessment is essential.

Future prospective studies using standardized imaging protocols, microbiological confirmation, and longitudinal follow-up are needed to clarify the impact of tuberculosis-related changes on lung cancer staging, treatment decisions, and patient outcomes.

## Figures and Tables

**Table 1:** Demographic, clinical, and laboratory characteristics of the study population.

		Mean	SD	95.0% Lower CL for Mean	95.0% Upper CL for Mean	N	N %
Age (years)		67	10	66	68		
Sex	Female					267	43.1%
	Male					353	56.9%
Urban	No					249	40.2%
	Yes					371	59.8%
Hb g/dL		9	3	9	10		
WBC × 10^3^/µL		10	4	10	10		
PLT × 10^3^/µL		323	133	313	334		
ESR (mm/h)		50	36	47	53		
FBG (mg/dL)		116	44	112	119		
AST (U/L)		30	17	28	31		
ALT (U/L)		24	26	21	27		
Urea (mg/dL)		47	27	45	49		
Creatinine (mg/dL)		1.09	0.57	1.04	1.13		
SpO_2_		91	9	90	91		
Systolic blood pressure		133	20	131	134		
Heart rate		80	29	77	82		
Smoker	No					190	30.6%
	Yes					430	69.4%
Pack Year Index		40	25	38	42		

SD—standard deviation; N—number of patients; %—percentage; Hb—hemoglobin; WBC—white blood cell count; PLT—platelet count; ESR—erythrocyte sedimentation rate; FBG—fasting blood glucose; AST—aspartate aminotransferase; ALT—alanine aminotransferase; Urea—blood urea level; Creatinine—serum creatinine; SpO_2_—peripheral oxygen saturation; COPD—chronic obstructive pulmonary disease; GOLD—Global Initiative for Chronic Obstructive Lung Disease classification; O_2_ therapy—oxygen therapy. BMI was not included due to incomplete anthropometric documentation across the retrospective dataset.

**Table 2:** Clinical, histopathological, and functional characteristics of the study population.

		N =	N %
TB status	No TB	232	37.4
	Post TB sequelae	337	54.4
	Active TB	51	8.2
Histopathological diagnosis	Squamous cell carcinoma	71	11.5
	Small cell carcinoma	32	5.2
	Undifferentiated carcinoma/lymphoma suspicion	13	2.1
	Inconclusive/biopsy in progress/no HP confirmation	20	3.2
	NSCLC, unspecified	89	14.4
	Adenocarcinoma	359	57.9
	Other non-malignant diagnosis	6	1.0
	Mesothelioma/carcinoma–mesothelioma suspicion	5	0.8
	Atypical cells/suspicious	25	4.0
Cancer stage	early stage	245	39.5
	advanced stage	375	60.6
COPD	No	241	38.9
	Yes	379	61.1
GOLD stage	None	241	38.9
	GOLD I	11	1.8
	GOLD II	98	15.8
	GOLD III	122	19.7
	GOLD IV	111	17.9
Spirometry *	Missing/not performed	147	24.6
	Normal spirometry	81	13.5
	Obstructive dysfunction	179	29.9
	Restrictive dysfunction	31	5.2
	Mixed dysfunction	139	23.2
	Non-specific/mild ventilatory impairment	21	3.5
Respiratory failure	No	352	56.8
	Yes	268	43.2
Heart failure	No	442	71.3
	Yes	178	28.7
Hypertension	No	295	47.6
	Yes	325	52.4

* Spirometry results were available and interpretable in 598 of 620 patients (96.5%); the remaining 22 patients had missing or unperformed spirometry assessments and are included in the ‘Missing/not performed’ category (total n = 147). Abbreviations: TB—tuberculosis; COPD—chronic obstructive pulmonary disease; GOLD—Global Initiative for Chronic Obstructive Lung Disease classification; NSCLC—non-small cell lung cancer, unspecified; HP—histopathological examination.

**Table 3:** Strength of association between radiological imaging findings and patient group classification.

Radiological Finding	Pearson χ^2^	df	*p*-Value	Cramer’s V	Strength
Nodular lesions/metastatic dissemination	1.102	2	0.576	0.042	Negligible
Mediastinal/hilar involvement and adenopathy	11.285	2	0.004	0.135	Weak
Pleural involvement	11.075	2	0.004	0.134	Weak
Emphysematous/chronic obstructive changes	0.685	2	0.71	0.033	Negligible
Pulmonary opacities/condensation	24.877	2	<0.001	0.2	Small-to-moderate
Pulmonary tumor/expansive mass	2.410	2	0.3	0.062	Negligible
Atelectatic and retractile changes	51.902	2	<0.001	0.289	Moderate
Fibrotic/interstitial/bronchial changes	185.574	2	<0.001	0.547	Strong
Cavitary and destructive lesions	79.712	2	<0.001	0.359	Moderate
Infectious/inflammatory changes	122.075	2	<0.001	0.444	Moderate-to-strong

**Table 4:** Logistic regression analysis of radiological predictors associated with post-TB sequelae and active tuberculosis.

TB Status ^a^		B	Std. Error	Wald	df	Sig.	Exp(B)	95% Confidence Interval for Exp(B)	
								Lower Bound	Upper Bound
Post TB sequelae	Intercept	9.257	1.587	34.033	1	<0.001			
	Age	−0.050	0.013	14.257	1	<0.001	0.952	0.927	0.976
	Pack Year Index	−0.002	0.006	0.115	1	0.735	0.998	0.987	1.010
	Hb	−0.076	0.068	1.236	1	0.266	0.927	0.811	1.059
	WBC	−0.059	0.033	3.256	1	0.071	0.943	0.884	1.005
	PLT	−0.001	0.001	1.269	1	0.260	0.999	0.997	1.001
	ESR	−0.009	0.004	4.763	1	0.029	0.991	0.984	0.999
	Female	−2.772	0.311	79.572	1	<0.001	0.063	0.034	0.115
	Male	0 ^b^	.	.	0	.	.	.	.
	Respiratory failure—No	1.612	0.359	20.209	1	<0.001	5.015	2.483	10.128
	Respiratory failure—Yes	0 ^b^	.	.	0	.	.	.	.
	Cancer stage-I	−0.067	0.472	0.020	1	0.887	0.935	0.371	2.360
	Cancer stage-II	−1.242	0.388	10.262	1	0.001	0.289	0.135	0.618
	Cancer stage-III	−0.518	0.349	2.200	1	0.138	0.596	0.301	1.181
	Cancer stage-IV	0 ^b^	.	.	0	.	.	.	.
	Dyspnea-none	−1.073	0.499	4.628	1	0.031	0.342	0.129	0.909
	Dyspnea-mild	−1.243	0.572	4.719	1	0.030	0.289	0.094	0.886
	Dyspnea-moderate	−1.962	0.561	12.232	1	<0.001	0.141	0.047	0.422
	Dyspnea-severe	0 ^b^	.	.	0	.	.	.	.
	Cough-none	0.316	0.401	0.621	1	0.431	1.372	0.625	3.013
	Cough-dry	1.741	0.354	24.238	1	<0.001	5.702	2.851	11.402
	Cough-productive	0 ^b^	.	.	0	.	.	.	.
	Hemoptysis—No	1.109	0.337	10.816	1	0.001	3.032	1.566	5.873
	Hemoptysis—Yes	0 ^b^	.	.	0	.	.	.	.
	Smoker—No	−0.233	0.312	0.559	1	0.455	0.792	0.430	1.460
	Smoker—Yes	0 ^b^	.	.	0	.	.	.	.
	COPD—No	−2.480	0.393	39.764	1	<0.001	0.084	0.039	0.181
	COPD—Yes	0 ^b^	.	.	0	.	.	.	.
	Nodular lesions/metastatic dissemination—No	−0.879	0.314	7.861	1	0.005	0.415	0.225	0.768
	Nodular lesions/metastatic dissemination—Yes	0 ^b^	.	.	0	.	.	.	.
	Mediastinal/hilar involvement and adenopathy—No	−1.018	0.354	8.258	1	0.004	0.361	0.180	0.723
	Mediastinal/hilar involvement and adenopathy—Yes	0 ^b^	.	.	0	.	.	.	.
	Pleural involvement—No	−0.869	0.350	6.184	1	0.013	0.419	0.211	0.832
	Pleural involvement—Yes	0 ^b^	.	.	0	.	.	.	.
	Emphysematous and chronic obstructive changes—No	0.583	0.348	2.808	1	0.094	1.792	0.906	3.546
	Emphysematous and chronic obstructive changes—Yes	0 ^b^	.	.	0	.	.	.	.
	Pulmonary opacities/condensation—No	−1.676	0.312	28.772	1	<0.001	0.187	0.101	0.345
	Pulmonary opacities/condensation—Yes	0 ^b^	.	.	0	.	.	.	.
Active TB	Intercept	0.978	2.629	0.138	1	0.710			
	Age	0.028	0.023	1.435	1	0.231	1.028	0.982	1.076
	Pack Year Index	−0.024	0.011	5.351	1	0.021	0.976	0.956	0.996
	Hb	0.193	0.104	3.440	1	0.064	1.212	0.989	1.486
	WBC	−0.086	0.050	2.947	1	0.086	0.918	0.832	1.012
	PLT	0.000	0.002	0.057	1	0.812	1.000	0.997	1.004
	ESR	0.014	0.006	4.895	1	0.027	1.014	1.002	1.026
	Female	−2.911	0.516	31.805	1	<0.001	0.054	0.020	0.150
	Male	0 ^b^	.	.	0	.	.	.	.
	Respiratory failure—No	0.348	0.591	0.346	1	0.556	1.416	0.444	4.515
	Respiratory failure—Yes	0 ^b^	.	.	0	.	.	.	.
	Cancer stage-I	−0.683	0.669	1.042	1	0.307	0.505	0.136	1.874
	Cancer stage-II	−2.947	0.788	13.979	1	<0.001	0.052	0.011	0.246
	Cancer stage-III	−1.573	0.576	7.455	1	0.006	0.207	0.067	0.642
	Cancer stage-IV	0 ^b^	.	.	0	.	.	.	.
	Dyspnea-none	−1.977	0.847	5.441	1	0.020	0.139	0.026	0.729
	Dyspnea-mild	−1.614	0.962	2.815	1	0.093	0.199	0.030	1.312
	Dyspnea-moderate	−1.962	0.961	4.170	1	0.041	0.141	0.021	0.924
	Dyspnea-severe	0 ^b^	.	.	0	.	.	.	.
	Cough-none	−0.997	0.698	2.038	1	0.153	.369	0.094	1.450
	Cough-dry	−0.138	0.550	0.063	1	0.801	.871	0.296	2.559
	Cough-productive	0 ^b^	.	.	0	.	.	.	.
	Hemoptysis—No	−0.797	0.604	1.743	1	0.187	0.450	0.138	1.471
	Hemoptysis—Yes	0 ^b^	.	.	0	.	.	.	.
	Smoker—No	−2.486	0.669	13.827	1	<0.001	0.083	0.022	0.309
	Smoker—Yes	0 ^b^	.	.	0	.	.	.	.
	COPD—No	−0.474	0.569	0.692	1	0.405	0.623	0.204	1.901
	COPD—Yes	0 ^b^	.	.	0	.	.	.	.
	Nodular lesions/metastatic dissemination—No	0.699	0.476	2.155	1	0.142	2.012	0.791	5.117
	Nodular lesions/metastatic dissemination—Yes	0 ^b^	.	.	0	.	.	.	.
	Mediastinal/hilar involvement and adenopathy—No	−1.840	0.501	13.484	1	<0.001	0.159	0.059	0.424
	Mediastinal/hilar involvement and adenopathy—Yes	0 ^b^	.	.	0	.	.	.	.
	Pleural involvement—No	−0.202	0.545	0.137	1	0.711	0.817	0.281	2.379
	Pleural involvement—Yes	0 ^b^	.	.	0	.	.	.	.
	Emphysematous and chronic obstructive changes—No	2.106	0.550	14.644	1	<0.001	8.213	2.793	24.150
	Emphysematous and chronic obstructive changes—Yes	0 ^b^	.	.	0	.	.	.	.
	Pulmonary opacities/condensation—No	−0.761	0.490	2.407	1	0.121	0.467	0.179	1.222
	Pulmonary opacities/condensation—Yes	0 ^b^	.	.	0	.	.	.	.

^a^ The reference category is No TB; ^b^ This parameter is set to zero because it is redundant.

## Data Availability

Data is available on request to the corresponding author.
